# *Taisho-Sanshoku* koi have hardly faded skin and show attenuated melanophore sensitivity to adrenaline and melanin-concentrating hormone

**DOI:** 10.3389/fendo.2022.994060

**Published:** 2022-12-22

**Authors:** Yukari Shinohara, Satoshi Kasagi, Noriko Amiya, Yukihiro Hoshino, Ryo Ishii, Noriyuki Hyodo, Hiroaki Yamaguchi, Shoh Sato, Masafumi Amano, Akiyoshi Takahashi, Kanta Mizusawa

**Affiliations:** ^1^ School of Marine Biosciences, Kitasato University, Sagamihara, Kanagawa, Japan; ^2^ Niigata Prefectural Inland Water Fisheries Experiment Station, Ogawaramachi, Nagaoka, Niigata, Japan

**Keywords:** melanophore, koi carp, melanin-concentrating hormone, catecholamine, skin color, background color

## Abstract

**Introduction:**

Koi carp, an ornamental fish derived from the common carp *Cyprinus carpio* (CC), is characterized by beautiful skin color patterns. However, the mechanism that gives rise to the characteristic vivid skin coloration of koi carp has not been clarified. The skin coloration of many teleosts changes in response to differences in the background color. This change in skin coloration is caused by diffusion or aggregation of pigment granules in chromatophores and is regulated mainly by sympathetic nerves and hormones. We hypothesized that there would be some abnormality in the mechanism of skin color regulation in koi carp, which impairs skin color fading in response to background color.

**Methods:**

We compared the function of melanin-concentrating hormone (MCH), noradrenaline, and adrenaline in CC and *Taisho-Sanshoku* (TS), a variety of tri-colored koi.

**Results and Discussion:**

In CC acclimated to a white background, the skin color became paler and pigment granules aggregated in melanophores in the scales compared to that in black-acclimated CC. There were no clear differences in skin color or pigment granule aggregation in white- or black-acclimated TS. The expression of *mch1* mRNA in the brain was higher in the white-acclimated CC than that in the black-acclimated CC. However, the expression of *mch1* mRNA in the brain in the TS did not change in response to the background color. Additionally, plasma MCH levels did not differ between white- and black-acclimated fish in either CC or TS. *In vitro* experiments showed that noradrenaline induced pigment aggregation in scale melanophores in both CC and TS, whereas adrenaline induced pigment aggregation in the CC but not in the TS. *In vitro* administration of MCH induced pigment granule aggregation in the CC but not in the TS. However, intraperitoneal injection of MCH resulted in pigment granule aggregation in both CC and TS. Collectively, these results suggest that the weak sensitivity of scale melanophores to MCH and adrenaline might be responsible for the lack of skin color change in response to background color in the TS.

## 1 Introduction

Generally, teleosts can change their skin color in response to light intensity of the background (1, 2). When melanin granules inside melanophores aggregate, the skin color becomes paler, whereas when melanin granules diffuse, the skin color becomes darker (3). The endocrine and sympathetic nervous systems modulate these background color responses. Melanin-concentrating hormone (MCH), which is synthesized in the hypothalamus and secreted into the blood *via* the pars nervosa of the pituitary gland, stimulates pigment aggregation and lightens skin color (1). In contrast, melanophore-stimulating hormone (α-MSH) acts on melanophores to diffuse pigment granules and induces a darker color background (1). In general, the secretion of MCH is increased on a white background, which appears to be the primary hormonal regulator of pigment movement responding to the background color in teleosts (4). Furthermore, MCH synthesis is enhanced over a long-term acclimation to the white background color, and skin color remains paler (1). Additionally, noradrenaline secreted from the axon terminal bulge of sympathetic nerves and adrenaline secreted from chromaffin cells induces a rapid pigment translocation through binding with specific receptors (2, 5). Chromatic information of background is captured by the ventral and dorsal retinas, processed in the optic tectum and partly at the level of the motoneurons in the medulla, and sent to chromatophores *via* direct nervous connections (2, 3).

The Japanese koi carp are ornamental fish bred from a skin-color mutant of the common carp *Cyprinus carpio* (CC). Dozens of varieties have been produced as a result of approximately 200 years of selective breeding (6, 7). The skin color of CC is uniformly deep brown on the dorsal side, and like many other teleosts, the ventral skin color is paler than the dorsal side. Meanwhile, koi carp are characterized by their vivid color patterns: *Taisho-Sanshoku* (TS), also called *Sanke*, which has a white background with speckles of black and red; *Showa-Sanshoku*, also called *Showa*, which have white, black, and red markings, with a striking black pattern; and *Kohaku*, which have a white background with speckles of red and is the most famous variety (6). The skin color pattern of koi carp is expressed at the latest two months after hatching. In some koi varieties, skin color patterns may slowly change throughout life, but there are no differences in body coloration based on sex or sexual maturity. The more vivid colors in these patterns, the more valued the koi carp are. Therefore, if the koi carp change their skin color in response to the background color, the vividness of the color pattern, which is what is valued in koi carp, would decrease. As a result of selective breeding, the koi carp might be insensitive to changes in background color, making it more difficult for skin color to change paler. In preliminary experiments, we acclimated TS to a white background and found that the skin color hardly faded. Therefore, in the present study, we compared the functions of color regulators that promote skin color fading, i.e., melanin-concentrating hormone, noradrenaline, and adrenalin, in CC and the TS variety.

## 2 Materials and methods

### 2.1 Fish and rearing condition

All experimental fish was provided by the Niigata Prefectural Inland Water Fisheries Experiment Station (Nagaoka, Japan) in the present study. Before the start of the experiments, experimental fish were acclimated in black background tanks (42 cm wide × 90 cm deep × 44 cm height) for one week. During the acclimation period, the fish were fed commercial pellets (Idle; Kyorin, Himeji, Japan). The amount of food fed per day was limited to 1% of body weight (*BW*) to account for the possibility that differences in growth may affect skin color. Fish were fasted for 24 h prior to the start of the experiment. Indoor light was shut off and fluorescent lights were turned on from 6:00 to 18:00 every day. The photon flux density (PFD) was measured using a spectrometer USB4000 (Ocean Insight, Orlando, FL, USA). The PFD at the water surface was approximately 50 µmol m^-2^ s^-1^ and the water temperature was maintained at 25–28°C.

### 2.2 Background color adaptation and skin color analysis

To examine short- and long-term effects of background color adaptations on skin color regulation, CC and TS were kept in black- or white-colored tanks for 3 days or 3 weeks. Each period experiment was conducted using four tanks of identical size (42 cm wide × 90 cm deep × 44 cm height): two with a black background with a black bottom and sides made of plastic plates, and the other two with a white background with a white bottom and sides. For the 3-day experiment, 5-months old immature CC (38.8–105.8 g *BW*) and TS (18.8–69.1 g *BW*) were used. 3-month CC (14.3–25.2 g *BW*) and TS (4.4–11.8 g *BW*) were used for the 3-week experiment. During the acclimation period, 20 CC and 20 TS were kept in two separate black background tanks. After the acclimation, 20 CC were divided into two groups: 10 CC were moved into the other black tank, and the other 10 CC were moved into a white background tank. As with CC, TS was divided into two groups: 10 TS were moved into the other black background tank, and 10 TS were moved into a white background tank. During the experimental black or white background color adaptation, lighting, water temperature, feeding rate, and feeding frequency were the same as during the acclimation period. CC were identified by cutting a slit in the unpaired fin at the beginning of the experiment, whereas difference in color pattern was used to identify TS. The tanks were cleaned daily during the rearing period. No food residue or dead fish were observed during the rearing period.

Sampling was conducted 3 days and 3 weeks after the start of the rearing experiment. Blood was collected from all individuals under anesthesia with 0.1% 2-phenoxyethanol. Blood was centrifuged at 4°C at 3,000 g for 15 min, and the supernatant, i.e., plasma, was collected and stored at −80°C. Brains were collected and flash-frozen on a metal rack placed on dry ice and stored at −80°C. Specific growth ratio (*SGR*) and condition factor (*CF*) were calculated using the following equations:


$$SGR=\frac{\ln\left\{Final\;BW\;\left(g\right)\right\}-\ln\left\{Initial\;body\;BW\;\left(g\right)\right\}}{experimental\;period\;\left(days\right)}\times 100\;\left(\%\right)$$

$$CF=\frac{Final\;BW\;\left(g\right)}{{\left\{Final\;SL\;\left(cm\right)\right\}}^{3}}\times 100$$

The dorsal surface of each individual was photographed using a digital STR camera D3400 (Nikon, Tokyo, Japan) before and after rearing in the background color tanks. The skin lightness on each individual’s dorsal surface was measured using ImageJ (National Institutes of Health, Bethesda, MD, USA). All dorsal area anterior to the dorsal fin was used for skin color analysis in CC, and All black patches anterior to the dorsal fin were used for skin color analysis in TS (**Supplementary Figure 1**). The mean gray value in each analyzed area was calculated and used as a parameter for skin color lightness. The degree of skin color fading was calculated using the following equation:


$$Skin\;color\;fading=\frac{Final\;skin\;color\;lightness}{Initial\;skin\;color\;lightness}$$

### 2.3 Brain mRNA analysis

#### 2.3.1 Total RNA extraction and purification

Total RNA of the brain was extracted using ISOGEN II (Nippon Gene, Tokyo, Japan). Total RNA was treated with RNase-free DNase (TaKaRa, Otsu, Japan) to eliminate genomic DNA contamination and then purified by phenol-chloroform extraction, followed by isopropanol precipitation. RNA concentration was measured using NanoDrop 2000 (Thermo Fisher Scientific, Waltham, MA, USA) and RNA samples were stored at −80°C.

#### 2.3.2 cDNA cloning and reference RNA synthesis of *mch*

Primers were designed using the following carp reference sequences from the National Center for Biotechnology Information (NCBI) database (https://www.ncbi.nlm.nih.gov/nucleotide/): *mch1* (MG214480), *mch2a* (XM_019093067.2), and *mch2b* (LOC109077492) (**Supplementary Table 1**). Primer synthesis was outsourced to Eurofins Genomics (Tokyo, Japan). cDNA fragments of *mch1*, *mch2a*, and *mch2b* were amplified by reverse transcription PCR (RT-PCR) of RNA samples using a PrimeScript™ OneStep RT-PCR Kit (TaKaRa). Briefly, PCR reactions were performed in total volume of 20 µL, which was comprised of 1 µL of RNA, 7.4 µL of MilliQ water, 10 µL of 2×One step RT-PCR Buffer III, 0.4 µL of 10 µM forward primer, 0.4 µL of 10 µM reverse primer, 0.4 µL of TaKaRa Ex Taq HS, and 0.4 µL of Enzyme Mix II RT-PCR was performed using TaKaRa PCR Thermal Cycler Dice^®^ Touch (TaKaRa). The PCR cycling conditions were as follows: reverse transcription at 42°C for 5 min, followed by amplification; 95°C for 1 min, 40 cycles of 95°C for 20 s, 55–61°C for 20 s, and 72°C for 40 s.

PCR products were electrophoresed in Tris-acetate-EDTA buffer using a 2% agarose gel (Nippon Gene, Tokyo, Japan) to determine the molecular weight of the amplified products and then purified using the NucleoSpin Gel and PCR-Clean Kit (TaKaRa). Using the pGEM-T Easy Vector System (Promega, Madison, WI, USA), purified PCR was incorporated into a plasmid and transformed into ECOS™ Competent *Escherichia coli* XL1-Blue (Nippon Gene). After incubation at 37°C overnight on Luria-Bertani (LB) agar-100 μg/mL ampicillin, selected colonies were picked up and cultured in LB-100 μg/mL ampicillin at 37°C overnight. Plasmid DNA was then purified by the standard alkaline-SDS plasmid preparation method and sequenced using the BigDye Terminator v3.1 Cycle Sequencing Kit (Applied Biosystems, Foster City, CA, USA) and an ABI 3130xl genetic analyzer (Applied Biosystems).

Plasmid DNA was linearized using the appropriate restriction enzymes (*Sph*I, *Nco*I, or *Pst*I) (TaKaRa) and purified by phenol-chloroform extraction and isopropanol precipitation. T7 RNA polymerase or SP6 RNA polymerase (TaKaRa) was used to synthesize RNA fragments, using the DNA insert of the linearized plasmid as a template. Synthesized RNA was subjected to DNase treatment using recombinant RNase-free DNase I (TaKaRa) and then purified by acid phenol-chloroform treatment and isopropanol precipitation. Concentration of purified RNA samples was determined using NanoDrop 2000. The samples were then stored at −80°C as stepwise 10-fold dilutions to be used as reference RNA for quantitative reverse-transcription PCR (qRT-PCR).

#### 2.3.3 qRT-PCR of MCH mRNA

Primers and TaqMan probes for qRT-PCR were designed and prepared by Eurofins (**Supplementary Table 1**). qRT-PCR for *mch1* was performed using One-Step PrimeScript™ III RT-qPCR Mix (TaKaRa). One µL of brain RNA (1 ng/µL, see Section 2.3.1) or reference RNA (see Section 2.3.2), 3.4 µL of Milli-Q water, 5 µL of 2×One Step PrimeScript III RT-qPCR Mix, 0.2 µL of 10 µM forward primer, reverse primer, and TaqMan probe was mixed for a total reaction volume of 10 µL. qRT-PCR was performed using a LightCycler^®^ 96 (Roche, Basel, Switzerland). The PCR cycling conditions were as follows: reverse transcription at 42°C for 5 min, followed by amplification; 95°C for 1 min, 50 cycles of 95°C for 15 s, 59°C for 15 s, and 72°C for 15 s. qRT-PCR for *mch2a* and *mch2b* was performed using OneStep RT-PCR Kit (QIAGEN, Hilden, Germany). One µL of brain RNA or reference RNA, 5.45 µL of Milli-Q water, 2 µL of 5×One-step RT-PCR Buffer, 0.25 µL of 10 µM forward primer, reverse primer, and TaqMan probe, and 0.4 µL of dNTP mix and Enzyme mix were mixed for a total reaction volume of 10 µL. qRT-PCR was performed using a LightCycler 96. The PCR cycling conditions were as follows: reverse transcription at 42°C for 5 min, followed by amplification; 95°C for 1 min, 60 cycles of 95°C for 15 s, 58°C for 15 s, and 72°C for 15 s. The amount of mRNA of each *mch* gene in the brain RNA samples was quantified based on the amplitude scaled to the dilution series of the reference RNA for each *mch* gene. The mRNA levels of the genes in each sample were quantified based on the amplitude scaled to the dilution series of the reference RNA (typically 10^-1^–10^4^ fg) for each gene.

### 2.4 Time-resolved fluoroimmunoassay for plasma MCH

To 100 µL of plasma sample (see Section 2.2), 1 mL of 0.1% HCl was added, and the mixture was stirred for 10 min using a Direct Mixer DM-301 (AS ONE, Osaka, Japan). The mixtures were then centrifuged at 4°C at 17,700 g for 30 min and 900 µL of the supernatant was collected. Plasma samples were not obtained from two of the ten TS individuals acclimated to the black background (TSb). Using six of the eight plasma samples, three new samples were prepared by combining 50 µL of plasma samples from two individuals to obtain a total volume of 100 µL, whereas the remaining two samples were used as such (n = 5). Similarly, plasma samples were not obtained from two of the ten TS individuals acclimated to the white background (TSw). Using four of the eight plasma samples, two new samples were prepared by combining 50 µL of plasma samples from two individuals to obtain a total volume of 100 µL, whereas the remaining four plasma samples were used as such (n = 6). Time-resolved fluoroimmunoassay was used to measured plasma MCH concentration as described previously by Amiya et al. (8). Carp MCH1 synthesized by PH Japan (Hiroshima, Japan) was used as a reference control.

### 2.5 Intraperitoneal injection of MCH and *ex vivo* administration of MCH to saline-cultured scales

Eighteen immature CC (19.0–44.2 g *BW*) and TS (3.4–19.1 g *BW*) were used for the MCH dosing experiment. After one week of rearing in a fluorescent light/black background tank, the MCH dosing experiment was conducted. The light/dark (L:D) conditions were = 12:12, with lighting hours between 6:00–18:00 with a photon flux density of 7.0 µmol m^-2^ s^-1^. The water temperature was 20–29°C. Commercial pellets (Idle) were fed once daily and water was changed 30 min after the start of feeding.

For intraperitoneal administration of MCH, synthesized MCH1 (see Section 2.4) dissolved in saline was used. After anesthesia with 0.05% 2-phenoxyethanol, MCH solution was administered using a Hamilton syringe, inserting the needle into the midline between the pelvic fins at a dose of 0.1 µg/g *BW* or 1 µg/g *BW* (0.1% of *BW*). Controls were administered saline (n = 6). One hour after administration, scales were collected, and the center of the scales was photographed using an Eclipse N*i* optical microscope (Nikon). The image of scales was processed using ImageJ (**Supplementary Figure 2**). Briefly, the image of scales was converted to 8-bit grayscale. Then, the 8-bit image was segmented into the areas of diffuse pigment in the melanophores on the scales (black) and the other areas (white). A circle with a diameter of 1 mm was selected at the center of the scale. The mean grey value, which ranged from 0 to 255, within the circle was then calculated. The extent of melanosome area (%) was calculated by dividing the mean gray value of the selected area by 2.55.

One CC (37.7 g *BW*) and three TS (5.6–12.0 g *BW*) were reared under a fluorescent light/black background color for at least one week. The rearing conditions were the same as those used in the intraperitoneal dosing experiment described above. Experimental fish were anesthetized with 0.05% 2-phenoxyethanol and their scales were collected and washed in 20 mL of saline. The scales of three TS were pooled into a single dish. The scales were then divided into five groups of three and incubated in 200 µL of saline in a 24-well plate for 1 h at 20°C. The scales were then transferred to 200 µL of saline or different concentrations of MCH in saline (0.01 nM, 0.1 nM, 10 nM, and 1000 nM) and incubated at 20°C for 1 h. After incubation, the center of the scales was photographed and the extent of melanosome area (%) was calculated as described above for the intraperitoneal administration experiment.

### 2.6 *Ex vivo* administration of noradrenaline and adrenaline to scales

Ten CC (38.8–108.1 g *BW*) and ten TS (18.8–69.1 g *BW*) were used for noradrenaline and adrenaline dosing experiment. The fish were reared in a black background tank for at least 14 days under the conditions described in Section 2.5. A total of thirteen scales, five from each of six randomly selected experimental fish, were collected without anesthesia to avoid any effects of the anesthetic agent on the sympathetic nervous system. The samples were divided into five test plots (n = 6). The centers of the scales were photographed using an inverted microscope BZ-x710 (Keyence, Osaka, Japan). The scales were then immersed in 200 µL of saline or different concentrations of noradrenaline and adrenaline in saline (100 nM, 1 µM, 10 µM, and 100 µM) and incubated at 20°C for 12 min. After incubation, the centers of the scales were photographed using an inverted microscope. The extent of melanosome area (%) was calculated as described above for the intraperitoneal administration experiment (see Section 2.5). Melanosome dispersion (%) was calculated using the following formula:


$$Melanosome\;dispersion=\frac{Final\;extent\;of\;melanosome\;area}{Initial\;extent\;of\;melanosome\;area}\times 100\;\left(\%\right)$$

### 2.7 qRT-PCR of MCH receptor in the scale

Eight CC (12.3–26.5 g *BW*) and eight TS (13.9–30.7 g *BW*) were used for qRT-PCR analysis of MCH receptor gene (*mchr*) mRNA in the scale. The fish were reared in a black background tank for 7 days under the conditions described in Section 2.5. Experimental fish were anesthetized with 0.05% 2-phenoxyethanol, and three scales were collected from the dorsal skin of CC and the black-, red-, and white-colored dorsal skin of TS. Total RNA was extracted and treated with DNase, as described above (see Section 2.3.1). cDNA cloning and reference RNA synthesis of *mchr* was conducted as described in Section 2.3.2 with slight modification. For cDNA cloning, primers were designed using the following carp reference sequences from the National Center for Biotechnology Information (NCBI) database: *mchr1a* (XM_019088135.1), *mchr1b* (XM_019082294.1), *mchr2S* (XM_019103446.1), and *mch2L* (XM_019120005.1) (**Supplementary Table 1**). qRT-PCR for each *mchr* was performed as described in Section 2.3.3 with slight modification using TB Green^®^ Prime Script™ PLUS RT-PCR Kit (TaKaRa). One µL of scale RNA (10 ng/µL) or reference RNA, 3.6 µL of Milli-Q water, 5 µL of 2×One Step TB Green RT-PCR, 0.2 µL of 10 µM forward primer and reverse primer were mixed for a total reaction volume of 10 µL. qRT-PCR was performed using a Thermal Cycler Dice Real Time System II (TaKaRa). The PCR cycling conditions were as follows: reverse transcription at 42°C for 5 min, followed by amplification; 95°C for 10 s, 45 cycles of 95°C for 20 s, 58°C for 20 s, and 72°C for 20 s. The mRNA levels of the genes in each sample were quantified based on the amplitude scaled to the dilution series of the reference RNA (typically 10^-2^–10^3^ fg) for each gene.

### 2.8 RT-PCR of adrenalin receptor in the scale

Since there were as many as 16 subtypes of *adr*, we performed RT-PCR instead of qRT-PCR. Primers were designed using the following carp reference sequences from the NCBI database: *adra1-aa* (XM_042761647.1), *adra1-ab* (XM_042732354.1), *adra1-ba* (XM_019082829.2), *adra1-bb* (XM_042738804.1), *adra1-d* (XM_019078739.2), *adra2-a* (XM_042749474.1), *adra2-b1* (XM_019074399.2), *adra2-b2* (XM_042765420.1), *adra2-c* (XM_042716607.1), *adra2-da* (XM_019103100.2), *adra2-db* (XM_042748477.1), *adrb1* (XM_019113442.2), *adrb2-a* (XM_042738348.1), *adrb2-b* (XM_019114799.2), *adrb3-a*, (XM_019064976.2), *adrb3-b* (XM_042765371.1), and *β-actin* (M24113) (**Supplementary Table 1**). RT-PCR was performed as described in Section 2.3.2. Scale RNA from three individuals were mixed and used as templates. The PCR cycling conditions were as follows: reverse transcription at 42°C for 5 min, followed by amplification; 95°C for 1 min, 40 cycles of 95°C for 20. After electrophoresis of the RT-PCR products using 2% agarose gel (Nippon Gene), the ethidium bromide-stained gels were photographed using Printgraph Classic (Atto, Tokyo, Japan).

### 2.9 Statistical analysis

The statistical analysis software R version 4.1.0 (R Core Team, Vienna, Austria) was used for statistical analysis. All data are expressed as the mean ± standard error (SE). The Welch test was used to test for differences in mean values due to differences in background color. The mean difference between different doses of MCH, adrenaline, or noradrenaline was analyzed using one-way analysis of variance (ANOVA), followed by Tukey’s honestly significant difference (HSD) test. The mean difference between skin color was also analyzed using one-way ANOVA, followed by Tukey’s HSD test. Statistical significance was accepted at *P*-values less than 0.05.

## 3 Results

### 3.1 The skin color hardly faded in TS by white-background acclimation

In both the 3-day and 3-week rearing experiments, the mean *BW*, *SL*, and *CF* in both CC and TS groups decreased, and *SGR* was negative (**Supplementary Table 2**). TSw group shows significantly lower final *CF* and *SGR* than TSb (*P* = 0.018 and 0.004, respectively, *n* = 10). None of the experimental fish died during the study period.

**Figure 1:**
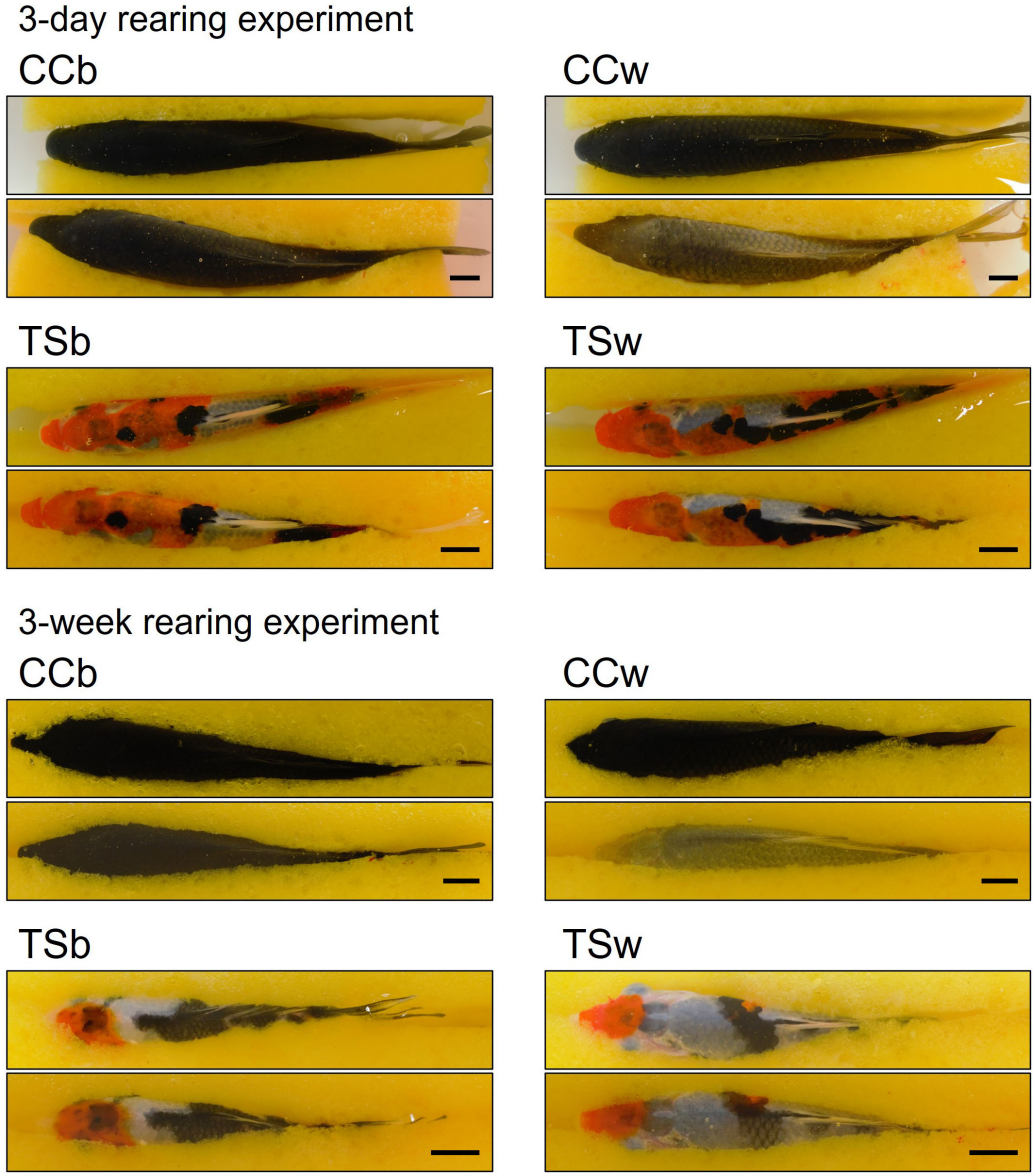
Effects of background color on skin color. Initial (upper) and final (lower) images of the dorsal skin of representative fish in 3 day- or 3 week-experiment. CCb, black-acclimated common carp; CCw, white-acclimated common carp; TSb, black-acclimated *Taisho-Sanshoku*; TSw, white-acclimated *Taisho-Sanshoku*. Scale bar = 1 cm.

**Figure 2:**
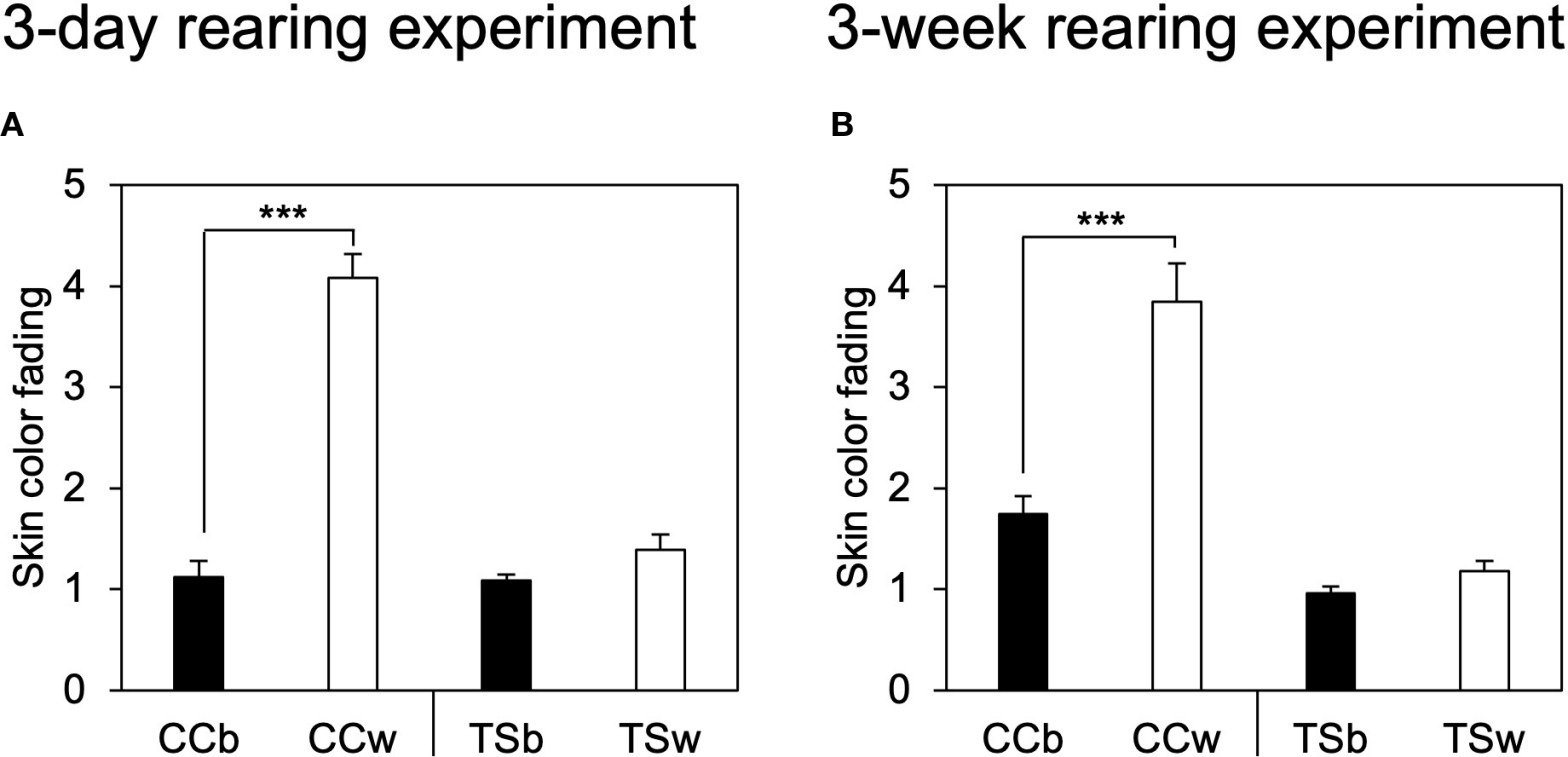
Effects of background color on skin color fading. Degree of skin color fading of common carp and *Taisho-Sanshoku* in the 3-day **(A)** and 3-week **(B)** rearing experiments. Data are shown as mean ± standard error (SE). Asterisks indicate statistically significant differences estimated using the Welch test (***P < 0.001, *n* = 10).

**Figure 3:**
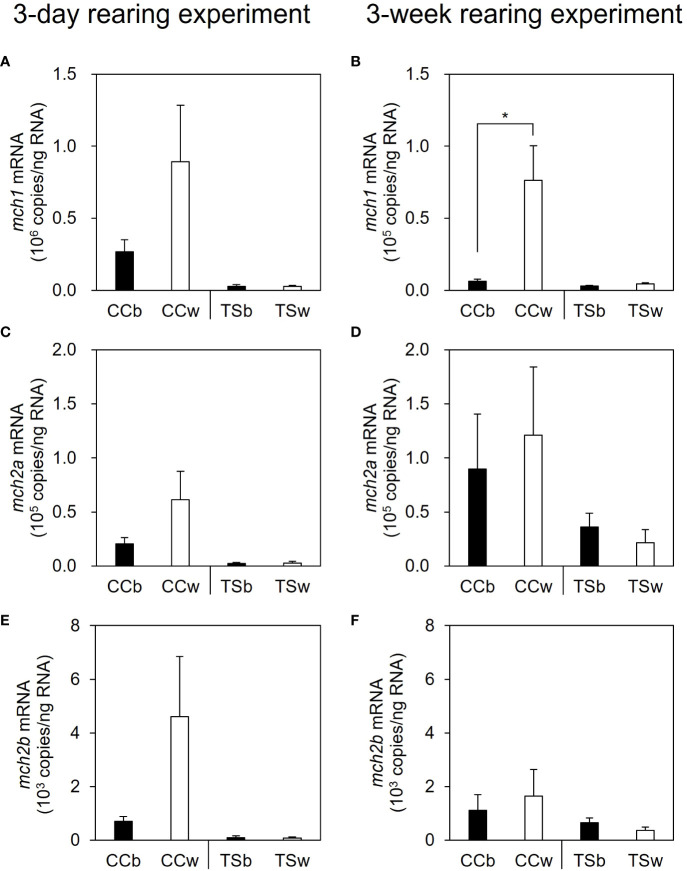
Effects of background color on the expression of melanin-concentrating hormone (*mch)* mRNA in the brain. The mRNA levels of *mch1*, *mch2a*, and *mch2b* in the brain in the 3-day **(A, C, E)** and 3-week rearing experiments **(B, D, F)**. Data are shown as mean ± SE. Asterisk indicates statistically significant difference estimated using the Welch test (*P < 0.05, *n* = 10).

**Figure 1** shows the representative dorsal skin images of CC and TS before and after 3-day and 3-week rearing experiment. The skin color of white-acclimated CC (CCw) transferred from a black background tank to a white background tank became significantly paler in the 3-day (*P* = 0.00000003, *n* = 10, **Figure 2A**) and 3-week rearing experiment (*P* = 0.0004, *n* = 10, **Figure 2B**). However, the skin color of TSw transferred from a black background tank to a white background tank did not change. Black-acclimated CC (CCb) and TSb that were transferred from a black background to a different black background tank also did not show a change in skin color. Similar results were obtained in the 3 week-rearing experiment (**Figure 2B**).

### 3.2 Brain *MCH* mRNA levels hardly changes with background color in TS

In the 3-day rearing experiment, no significant difference was observed in *mch1* expression in the brain between the CC and TS groups, in response to the background color (**Figure 3A**). In contrast, *mch1* expression in the brain was significantly higher in the CCw group than in the CCb group (*P* = 0.021, *n* = 10), while no significant difference in *mch1* expression in the brain was observed between the TSb and TSw groups in the 3-week rearing experiment (**Figure 3B**). Similarly, no significant difference in *mch2a* and *mch2b* expression in the brain was observed in the 3-day or 3-week rearing experiments, in response to the background color (**Figures 3C–F**).

### 3.3 Plasma MCH concentration hardly changes with background color in CC and TS

**Figure 4:**
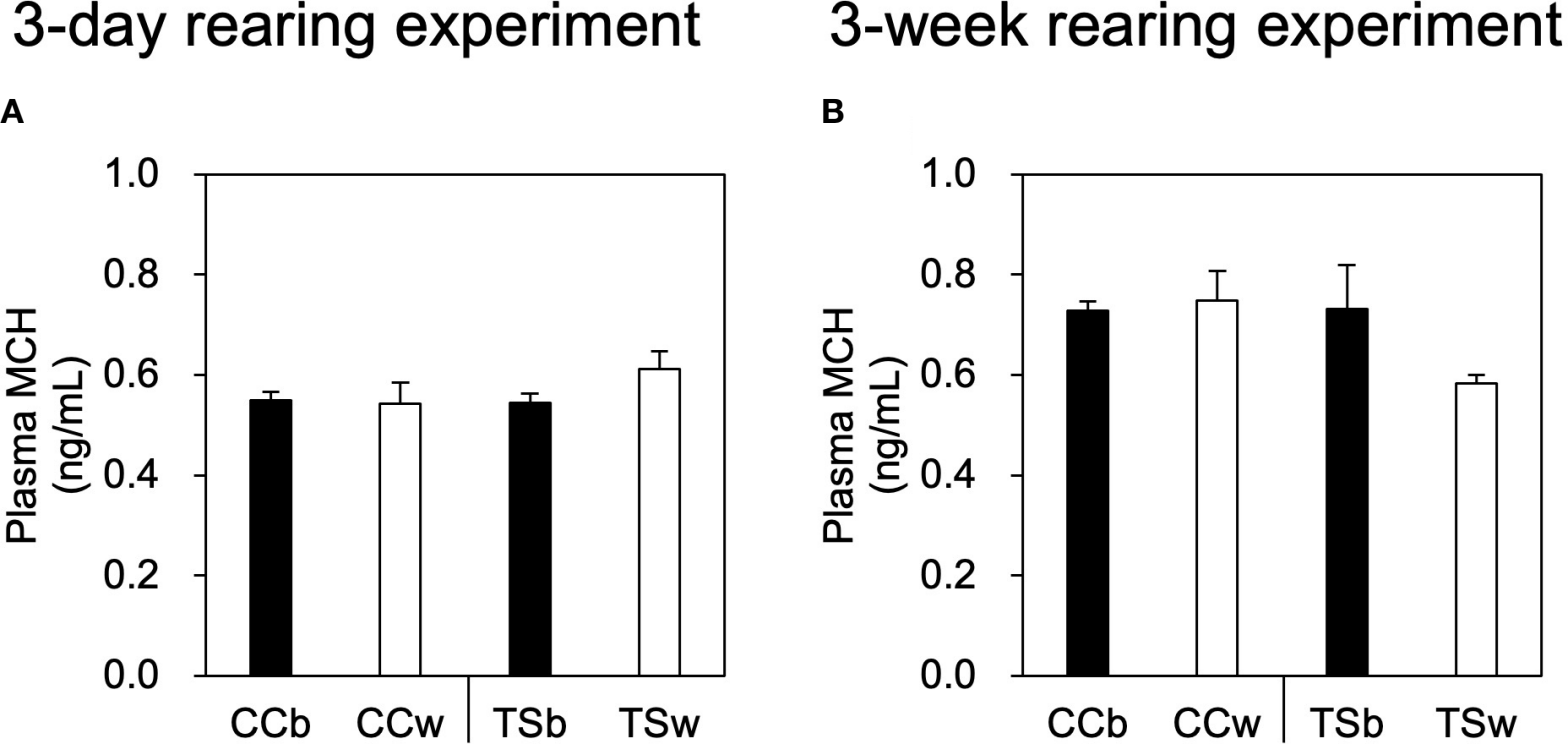
Effects of background color on plasma MCH level. Plasma MCH levels in the 3-day rearing experiment **(A)** and 3-week rearing experiment **(B)**. Data are shown as mean ± SE. No significant difference was estimated using the Welch test; *n* = 10 for CCb and CCw *n* = 5 for TSb, and *n* = 6 for TSw.

There were no significant differences in the plasma MCH concentrations of CC and TS due to differences in background color in both the 3-day and 3-week rearing experiments (**Figure 4**).

### 3.4 *In vivo* and *ex vivo* MCH administration induces melanosome aggregation

**Figure 5:**
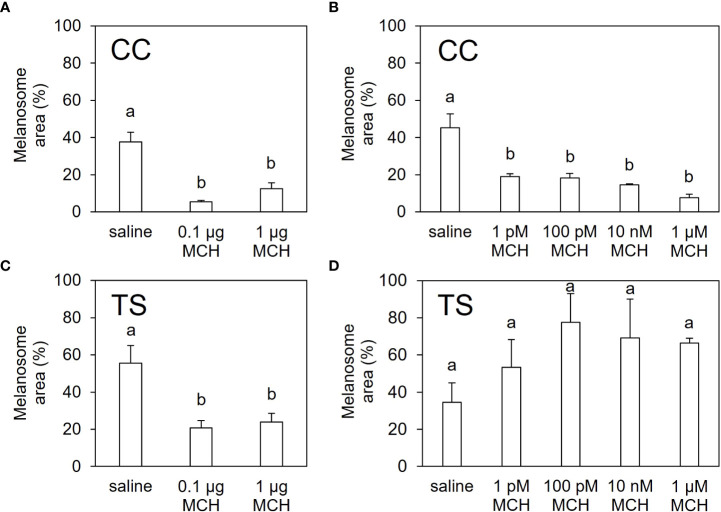
*In vitro* and *ex vivo* pigment-aggregating effects of MCH. Extent of melanosome area in the scales of CC **(A, B)** and TS **(C, D)** injected intraperitoneally with MCH **(A, C)** or treated with MCH *ex vivo* **(B, D)**. Data are shown as mean ± SE. Different letters indicate statistically significant differences estimated by one-way analysis of variance (ANOVA) followed by Tukey’s honestly significant difference (HSD) test; *P* < 0.05, *n* = 6 for **(A)** and **(C)**, *n* = 3 for **(B)** and **(D)**.

Intraperitoneal injection of MCH significantly decreased the melanosome area on scales from both the CC and TS groups (**Figures 5A, C**; **Supplementary Figure 3**). In *ex vivo* experiments, the melanosome area of the scales from the CC group decreased following MCH administration (**Figure 5B**; **Supplementary Figure 4**). In contrast, no significant changes in the melanosome area were observed in scales from the TS group (**Figure 5D**; **Supplementary Figure 4**).

### 3.5 *Ex vivo* noradrenaline/adrenaline administration induces melanosome aggregation

**Figure 6:**
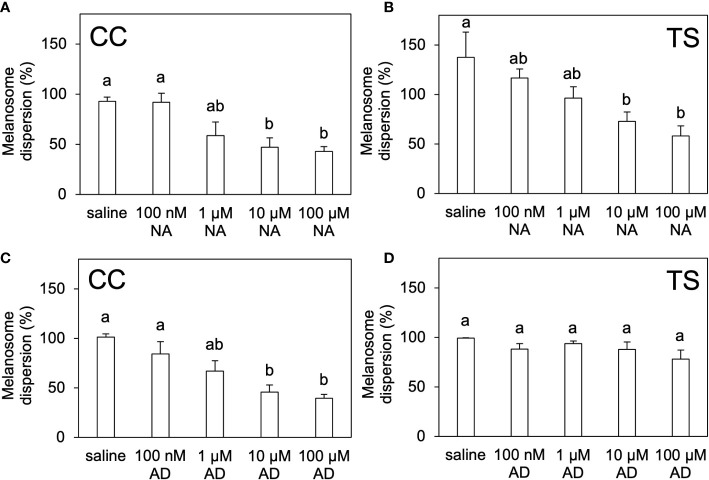
*In vitro* pigment-aggregating effects of noradrenaline and adrenaline. Degree of melanosome dispersion in the scales of CC **(A, C)** and TS **(B, D)** treated *ex vivo* with noradrenaline **(A, B)** or adrenaline **(C, D)**. Data are shown as mean ± SE.Different letters indicate statistically significant differences estimated by one-way ANOVA followed by Tukey’s HSD test; *P* < 0.05, *n* = 6.

The melanosome area in the saline-cultured scales of the CC and TS groups decreased following noradrenaline administration in a dose-dependent manner (**Figures 6A, B**; **Supplementary Figures 5**, **6**). Similarly, the melanosome area in the saline-cultured scales of the CC group decreased following adrenaline administration in a dose-dependent manner (**Figure 6C**; **Supplementary Figure 5**). However, the melanosome area in the saline-cultured scales of the TS group were not affected by adrenaline administration (**Figure 6D**; **Supplementary Figure 6**).

### 3.6 Expression levels of *mchr* in TS scales is equal to or greater than that in CC

**Figure 7:**
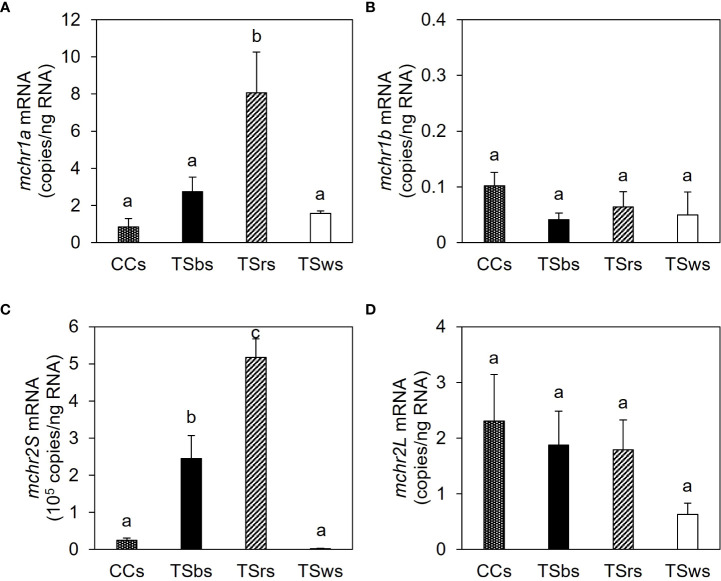
Expression of melanin-concentrating hormone receptor (*mchr)* mRNA in the scale. The mRNA levels of *mchr1a* **(A)**, *mchr1b* **(B)**, *mchr2S* **(C)**, and *mchr2L* **(D)** in the dorsal skin of common carp (CCs) and black-, red- and white-colored dorsal skin of TS (TSbs, TSrs, and TSws, respectively). Data are shown as mean ± SE. Different letters indicate statistically significant differences estimated by ANOVA followed by Tukey’s HSD test; *P* < 0.05, *n* = 8.

The mRNA contents of *mchr2S* were 0.2–5.2 × 10^5^ copies/ng total RNA in the scales of CC and TS. In contrast, the mRNA contents of *mchr1a*, *mchr1b*, and *mchr2L* were 0.8–8.1, 0.04–0.1, and 0.6–2.3 copies/ng total RNA, respectively (**Figure 7**). The *mchr2S* expression was highest in TS red scales, followed by TS black scales, and lower in TS white and CC scales. The *mchr1a* expression was highest in TS red scales and lower in TS black, TS white, and CC scales. No significant difference was observed in *mchr1b* and *mchr2L* between scales.

### 3.7 There are no apparent differences in the expression profile of *adr* in the scale of CC and TS

There was no apparent difference in the expression profile of *adr* subtypes between TS black, TS red, and CC scales (**Supplementary Figure 7**). The expression of *adra1-ab* was observed in the TS black, TS red, and CC scales but not in the TS white scales.

## 4 Discussion

Previous studies have shown that the skin color of the CC changes from darker to paler in white backgrounds and vice versa in dark backgrounds (9, 10). Consistent with these results, we observed that the skin color of the CC group became paler after 3 days and 3 weeks of rearing in a white background tank. In contrast, the skin color of the TS group did not change after 3 days or 3 weeks of rearing in a white background tank. This finding suggests a difference in the regulatory mechanism of skin color change in response to the background color between the CC and TS groups.

Because the skin color of the TS group did not fade under white background conditions, we examined the expression levels of three genes encoding MCH, which promotes skin color fading, in the brain. We found that *mch1* mRNA levels were higher in the CCw group than in the CCb group after 3 weeks of acclimation. However, there was considerable variation in expression levels among individuals, and no statistically significant differences were observed after 3days acclimation. These results suggested that *mch1* mRNA expression is promoted in a white background. However, an extended period of exposure to white background is needed for *mch1* mRNA levels to change in response to background color in the CC group. In contrast, in the TS group, there was no difference in *mch1* expression between the TSw and TSb groups after 3 weeks of acclimation, suggesting that the effect of background color on *mch1* expression in TS was limited.

The change in skin color in response to background color initially begins with the optical recognition of background color. In fish, the eye, responsible for vision, and the pineal gland, responsible for non-visual photoreception, are known to be required for the background color response of skin color (11, 12). The zebrafish pineal gland produces an antagonist of the melanocortin receptor, agouti-related peptide 2, which suppresses the expression of *mch1* and *mch2* in the hypothalamus (12). Although there is no evidence of a difference in the photoreceptor system of CC and TS to the authors’ knowledge, some changes in the retina and pineal gland may have occurred during the breeding process of TS, weakening the background color response in *mch*1 expression.

The expression levels of *mch2a* and *mch2b* in the brain did not differ significantly according to background color in the CC and TS groups, suggesting that the role of *mch2a* and *mch2b* in carp skin color regulation is limited. While *mch2* is found in all vertebrates, including fish, *mch1* is only found in teleosts (13). However, the role of MCH2 in skin color regulation in teleosts is still unclear. In zebrafish, overexpression of *mch2* induces skin color fading (14) and a brighter environment enhances *mch2* expression (12, 15), suggesting a role of *mch2* in skin color adaptation in response to background color. However, the response of *mch2* expression to changes in background color is limited when compared to *mch1*, indicating a more modest role for MCH2 in skin pigmentation (16). A comparably limited response of *mch2* to a white background has also been reported in starry flounders *Platichthys stellatus* (17). Thus, it seems likely that *mch1* plays a more important role than *mch2* in regulating skin coloration in teleosts.

Under the experimental conditions of this study, the CCw group showed paler skin color and higher *mch1* expression than the CCb group. In contrast, the TSw group did not show any skin color fading and no difference in *mch1* expression was observed compared to that in the CCb group. Therefore, we expected higher plasma concentrations of MCH1 in the CCw group than in the CCb group. Contrary to our expectation, there was no difference in plasma MCH levels in the CC groups depending on the background color. The MCH antibody used in this experiment was created using carp MCH1 as an antigen (epitope). However, it is unknown whether this antibody also binds to MCH2a or MCH2b; therefore, the MCH levels measured in this study were considered to represent the total amount of MCH1, MCH2a, and MCH2b. In the present study, even if plasma MCH1 levels in the CCw group were higher than in the CCb group, comparable levels of plasma MCH2a and MCH2b could mask the difference in total plasma MCH levels. In vertebrates, there are two subtypes of MCH receptors (MCHR), MCHR1 and MCHR2 (13). Although the ligand specificities of MCHs and MCHRs of CC are not yet known, there is no difference in the binding specificities of MCHR1 and MCHR2 to MCH1 and MCH2 in barfin flounder (18). Assuming that the ligand specificities in CC are similar to those in barfin flounder, the effect on pigment cells would also be equivalent. Based on this assumption, the difference in skin color between the CCw and CCb groups cannot be attributed to plasma MCH levels.

When MCH1 was administered to saline-cultured, dose-dependent pigment aggregation was observed in the scale melanophores in the CC group, but no aggregation was observed in the TS group. This result suggests that sensitivity in scale melanophores to MCH is lower in the TS group than that in the CC group. Therefore, even if MCH concentrations in the skin are equal, the pigment in melanophores in the TS group will be more dispersed than that in the CC group. As explained above, MCHRs have multiple subtypes. This study reveals that the significant MCHR gene expressed in CC scales is the *mchr2S*. In zebrafish, goldfish, and barfin flounder, *mchr2* but not *mchr1* is expressed in the skin (16, 19, 20). In zebrafish, *mchr2* regulates body color (14). Taken together, MCHR2S might function as the primary receptor for MCH in CC. The expression levels of *mchr2S* in TS black scales were not smaller than CC black scales, suggesting less sensitivity of TS melanophores to MCH is not due to the lack of the receptor.

Interestingly, *mchr2S* expression in TS was highest in red scales, followed by black scales. TS res scales contain xanthophores and iridophores but not melanophores (data not shown). Therefore, *mchr2S* might also be expressed in xanthophores. Alternatively, the MCHRs of TS and CC have differences in their ligand binding ability or signal transduction. Future analysis of MCHR may reveal the differences in MCH sensitivity at the receptor levels.

Interestingly, pigment aggregation was observed in both the CC and TS groups that were intraperitoneally injected with MCH. However, the direct effect of MCH on melanophores was weaker in the TS group than that in the CC group. Thus, intraperitoneal injection of MCH may act on melanophores by regulating the secretion of other skin color-regulating factors. α-MSH is involved in skin coloration in many vertebrates, including fish (1). In fish, α-MSH is produced and secreted in the pars intermedia of the pituitary gland and acts on chromatophores to promote pigment diffusion (1). The secretion of α-MSH from the pituitary gland of tilapia is inhibited by MCH (21). In CC, MCH nerves project to α-MSH-producing cells in the pituitary gland (22, 23). Therefore, MCH may suppress α-MSH in the pituitary glands of CC. In the present study, *mch1* expression in the brain was higher in the CCw group than in the CCb group; however, there was no apparent change in plasma MCH levels. Taken together, these results suggest that MCH may promote skin color fading in CC by suppressing pituitary secretion of α-MSH. Unfortunately, at present, we do not have a system for measuring α-MSH levels. In future studies, we hope to test this hypothesis by investigating α-MSH secretions from the pituitary gland and plasma α-MSH levels.

In fish, noradrenaline and adrenaline secreted by the sympathetic nervous system are essential for skin color regulation and promote rapid changes in skin color (24). When CC were transferred from a black background tank to a white background tank, an apparent and quick fading of skin color was observed in seconds to minutes (data not shown). This change could be attributed to noradrenaline or adrenaline in the sympathetic nervous system. In the present study, *ex viv*o administration of noradrenaline to saline-cultured scales induced melanosome aggregation in both the CC and TS groups at a similar concentration. Therefore, noradrenaline sensitivities of melanophores in both CC and TS groups may be comparable. As mentioned above, CC show a rapid change in skin color in response to changes in the background color, whereas TS shows little or no skin color change in the short or long term. These results suggest that the sympathetic nervous system of TS may be less responsive to changes in background color. Additionally, adrenaline only induced melanosome aggregation in the CC group. Weak adrenergic sensitivity of melanophores in TS may be one of the reasons why TS are less susceptible to skin color changes than CC. No apparent differences in the expression profiles of *adr* subtypes were observed in the scales of CC and TS, at least by RT-PCR analysis. Quantitative analysis by qRT-PCR is challenging because of the large number of structurally similar *adr* subtypes. Further analysis using transcriptome may help identify receptor subtypes involved in adrenergic sensitivity.

In conclusion, this study revealed that the sensitivity of melanophores to MCH and adrenaline is lower in TS than in CC. As both MCH and adrenaline promote pigment aggregation in CC, the impaired action of these skin color regulators may be the reason why TS are less susceptible to skin color fading than CC. Koi carp, including TS, have been bred as ornamental fish for approximately 200 years. As a result of selective breeding fish that show vivid contrasting patterns under various photic environments in the ponds of temples, shrines, and residences, phenotypes insensitive to changes in skin color in response to background color may have become fixed. Further comparative analysis of the skin color regulatory mechanism of Koi carp will hopefully reveal more variations in neural, endocrine, and chromatophore functions.

## Data availability statement

The raw data supporting the conclusions of this article will be made available by the authors, without undue reservation.

## Ethics statement

Ethical review and approval was not required for the animal study because all experiments were conducted following the Guidelines for the Care and Use of Animals at Kitasato University. No ethical review and approval are assigned because fish are not included in animal experiments under the regulation.

## Author contributions

All authors contributed to the study conception and design, acquisition, analysis or interpretation of data, drafting the article or revising it critically for important intellectual content. YS, SK, AT and KM designed the study. Data were collected by YS, NA, YH, RI, NH, HY, SS, MA and KM. Data analysis was performed by YS, NA, and KM. KM takes the final responsibility for this article. All authors contributed to the article and approved the submitted version.
