# Supplementary material for: Taisho-Sanshoku koi have hardly faded skin and show attenuated melanophore sensitivity to adrenaline and melanin-concentrating hormone
Source: Front Endocrinol (Lausanne). 2022 Dec 22;13:994060. doi: 10.3389/fendo.2022.994060 (PMC9813866; doi:10.3389/fendo.2022.994060)
Supplement: Supplementary file 1 [file DataSheet_1.pdf]

Supplementary Table 1. Primers and probes for reverse-transcription PCR and quantitative reverse-transcription PCR

Gene	Orien-tation	Nucleotide sequences (5'~3')	Tm (°C)	Amplicon size (bp)
Primers used for reverse-transcription PCR				
mch1	Forward	ATGAAGCTTTCCGTTGGTACTGTCCTCAT	61.3	375
	Reverse	CTACACTTCCCAGCATGGCCGGT	60.3	
mch2a	Forward	ATGGCATCTTCCTACATATTCATCATTGCAC	60.5	450
	Reverse	TCAAGCTTGCCAACAGGGCCTGTA	60.7	
mch2b	Forward	ATGATCATGGCATCATCTTACATGGTCATCT	60.8	471
	Reverse	TCAAGCTTGCCAGCAGGGCCT	61.1	
mchr1a	Forward	TTTTATATGTGTATTTTATTGTGTGCTTAGTGACC	58.1	1099
	Reverse	GTTGATCAATGCTTCAATTAGGTTGTTTTAAAG	58.2	
mchr1b	Forward	TCAAAGCACTGGAGGATTATCAGATAAAGTT	59.7	1159
	Reverse	TATTATAGACACAAAAAATGAGTTGAGCATGAGT	59.5	
mchr2S	Forward	AGCGTGCGAAGACCAGACTAAGA	59.7	1154
	Reverse	CATTTTCCTCAGTTAGCATGTCCAGTGA	59.4	
mchr2L	Forward	AACCTACCAGGACTTTCCATGGACATACAA	59.7	1192
	Reverse	GCTTATGCATTGATTCATCCATTTACAGTGTT	59.8	
adra1-aa	Forward	CTGGGAATGGTGTTGGCGAT	60.5	527
	Reverse	TACATCGACAGAATCACCACTAG	60.9	
adra1-ab	Forward	CTCTGTGGTGTGCCATCGC	61.6	645
	Reverse	AGCAGACGCATGAAGGCGAA	60.5	
adra1-ba	Forward	GACCGAACCGAGCTCGTTC	61.6	537
	Reverse	TAGAAGGAGCCCAGCGAGG	61.6	
adra1-bb	Forward	TTAAATGGCACAGAACTCGAGC	60.1	514
	Reverse	AAAAGGGTTCCTCGGTTATTACG	60.9	
adra1-d	Forward	TTGACTCTCAAGTCATCGGTGT	60.1	567
	Reverse	CCACAATGTATACTCTAGTGTACAT	60.9	
adra2-a	Forward	CCTTATTGTCTTTGGCAATGTGC	60.9	506
	Reverse	TCTGGTAAATTCGGACATACACG	60.9	
adra2-b1	Forward	ACGGGTCTGCATGGCCACA	61.6	533
	Reverse	CCTTTGTTCTTGTTCATGGAGAG	60.9	
adra2-b2	Forward	CATCTCTTTCCCCCCGCTG	61.6	654
	Reverse	ACGACCGCTAGGACGAATGT	60.5	
adra2-c	Forward	ATCCTCTGGGAATTCTACAAGTC	60.9	636

	Reverse	CGCTTTTCAGACATATTTCGGGT	60.9	
adra2-da	Forward	ATCCTCGTGGCCACGCTAG	61.6	578
	Reverse	CTCTCCTCCAGGTCGATATC	60.5	
adra2-db	Forward	TCATTCTCGTGGTTACCGTGAT	60.1	584
	Reverse	TCGCTCCATCCCGTTCTTAG	60.5	
adrb1	Forward	GGAGACGGTTTACCGTCCG	61.6	591
	Reverse	AATAGAGGAGGAGATGGCATATG	60.9	
adrb2-a	Forward	CCAACGACTTCAGACCGTCA	60.5	499
	Reverse	CCTTGCCTCCTGGAAGACG	61.6	
adrb2-b	Forward	TGATCTTAATTAGCATCTTAATGGTG	60.1	615
	Reverse	CTTCAGTGCGGTCAATTTTCTG	60.1	
adrb3-a	Forward	TGGCATGTGCTGACCTCATAAT	60.1	604
	Reverse	GAACTTTTCCGCCTTGCCGT	60.5	
adrb3-b	Forward	TTCTTCCTCTGGCACTGGTC	60.5	559
	Reverse	AACACCTTCCCGTACACAAACA	60.1	
b-actin	Forward	TGAAGTACCCCATCGAGCAC	53.2	377
	Reverse	AGGATCTTCATGAGGTAGTC	48.3	
Primers used for quantitative reverse-transcription PCR				
mch1	Forward	TGAAGACACGGAGCCAGATCT	71	97
	Reverse	GTCGGCCACCCGATGAT	69	
	Probe	AATGCTCTGAGATCCACACCAGGCAAC	74.3	
mch2a	Forward	CACATTCAGAAGACACCCCATCA	59	106
	Reverse	CCCTCCCAAGAGACCCTTTTA	58	
	Probe	AAACGCATCTTCATACTTGCCGACACAGAA	68	
mch2b	Forward	ACAGAAGGAGAAGACTATGTAAC	59	149
	Reverse	CTCCCAAGAGACCCTTTTATTC	60	
	Probe	AAACGCATCTTCATACTTGCTGACACAGGA	68	
mchr1a	Forward	TCGGAGCCACTATGTGCAAA	55	100
	Reverse	GCCACATAACGGTCCAAGGT	57	
mchr1b	Forward	GCTGATAATTCCAGTGATGAGTT	59.2	132
	Reverse	GATGCAGTTGCCAATTATACCGA	60.9	
mchr2S	Forward	TCTGGCCAACGGTTTGGT	55	283
	Reverse	CCTCTTTTCAGATGTAGGATGTACAATT	57	
mchr2L	Forward	CTGCAGTCATGACCGCTATGA	58	100
	Reverse	TTAATGCGGATTGTCCTGGAT	58	
