# Supplementary material for: Taisho-Sanshoku koi have hardly faded skin and show attenuated melanophore sensitivity to adrenaline and melanin-concentrating hormone
Source: Front Endocrinol (Lausanne). 2022 Dec 22;13:994060. doi: 10.3389/fendo.2022.994060 (PMC9813866; doi:10.3389/fendo.2022.994060)
Supplement: Supplementary file 2 [file DataSheet_2.pdf]

Supplementary Table 2. Growth parameters of common carp and Koi carp acclimated to black or white background.

	Initial *BW* (g)	Final *BW* (g)	Initial *SL* (cm)	Final *SL* (cm)	Initial *CF*	Final *CF*	*SGR*
3-day rearing experiment							
CCb	76.4 ± 6.5	74.9 ± 6.7	14.1 ± 0.4	14.1 ± 0.4	2.66 ± 0.06	2.57 ± 0.07	-0.12 ± 0.03
CCw	76.0 ± 5.6	74.4 ± 5.2	14.4 ± 0.4	14.3 ± 0.3	2.50 ± 0.08	2.49 ± 0.05	-0.10 ± 0.07
TSb	31.9 ± 4.2	30.2 ± 4.0	10.4 ± 0.4	10.4 ± 0.4	2.69 ± 0.05	2.56 ± 0.02	-0.29 ± 0.01**
TSw	32.1 ± 2.8	30.0 ± 2.6	10.5 ± 0.3	10.6 ± 0.3	2.73 ± 0.09	2.48 ± 0.03	-0.35 ± 0.01
3-week rearing experiment							
CCb	18.2 ± 1.0	18.0 ± 1.0	9.0 ± 0.2	9.3 ± 0.2	2.47 ± 0.05	2.23 ± 0.08	-0.05 ± 0.23
CCw	18.3 ± 0.7	17.9 ± 0.7	9.0 ± 0.1	9.0 ± 0.1	2.51 ± 0.12	2.45 ± 0.07	-0.09 ± 0.21
TSb	8.8 ± 0.6	8.7 ± 0.6	6.9 ± 0.2	6.9 ± 0.2	2.70 ± 0.06	2.63 ± 0.04*	-0.06 ± 0.03
TSw	8.7 ± 0.6	8.4 ± 0.6	7.0 ± 0.2	7.0 ± 0.2	2.54 ± 0.06	2.47 ± 0.04	-0.16 ± 0.05

Abbreviations: *BW*, body weght; *SL*, standard length; *CF*, condition factor; *SGR*, specific growth ratio; CCb, black-acclimated common carp; CCw, white-acclimated common carp; TSb, black-acclimated *Taisho-Sanshoku*; TSw, white-acclimated *Taisho-Sanshoku*. Asterisks represents a significant difference between black- and white-acclimated fish (Welch test, * $P < 0.05$, ** $P < 0.01$, $n = 10$)
