# Supplementary figures and images for: Taisho-Sanshoku koi have hardly faded skin and show attenuated melanophore sensitivity to adrenaline and melanin-concentrating hormone
Source: Front Endocrinol (Lausanne). 2022 Dec 22;13:994060. doi: 10.3389/fendo.2022.994060 (PMC9813866; doi:10.3389/fendo.2022.994060)

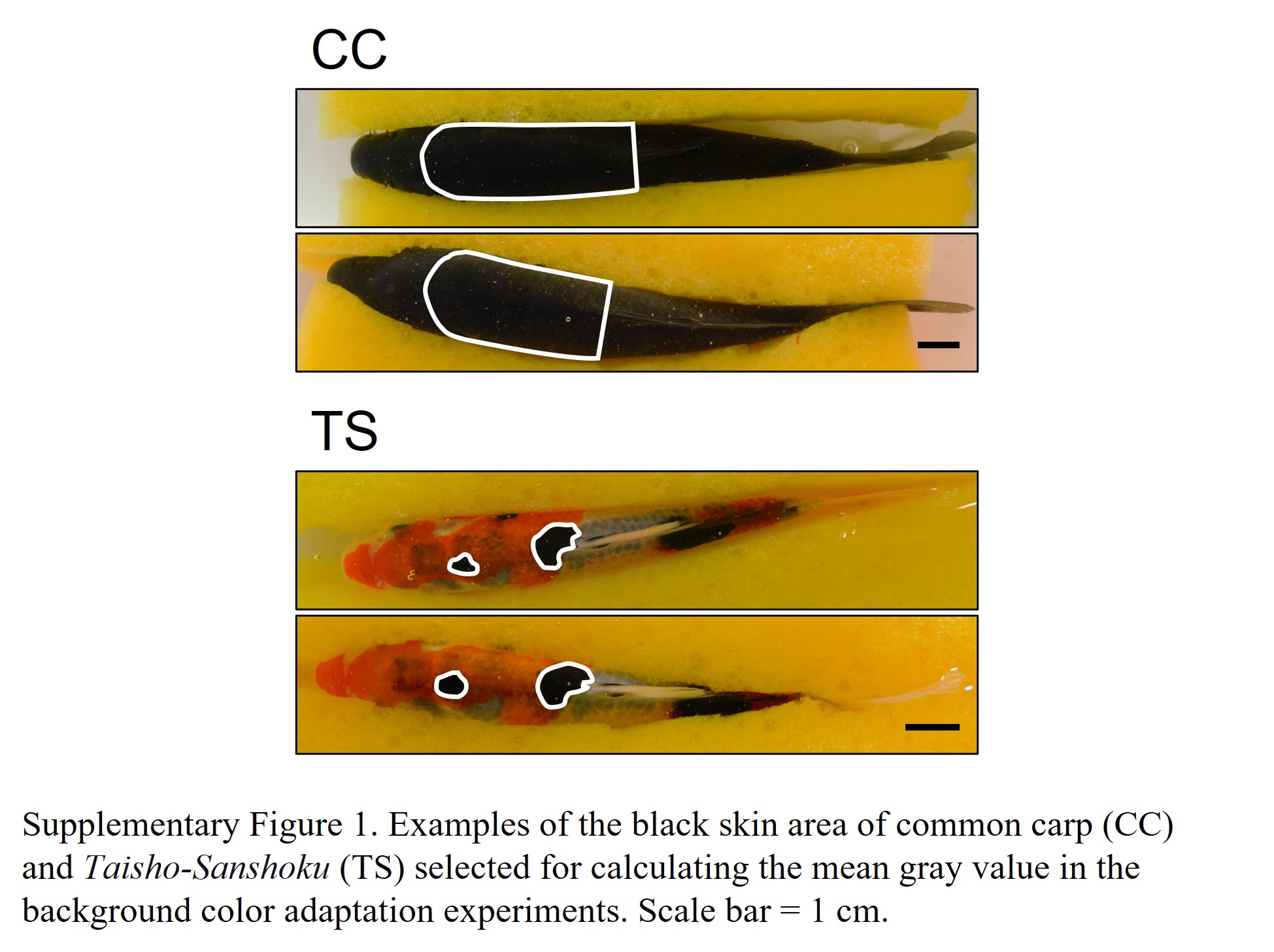

Supplement: Supplementary file 3 [file Image_1.jpeg]

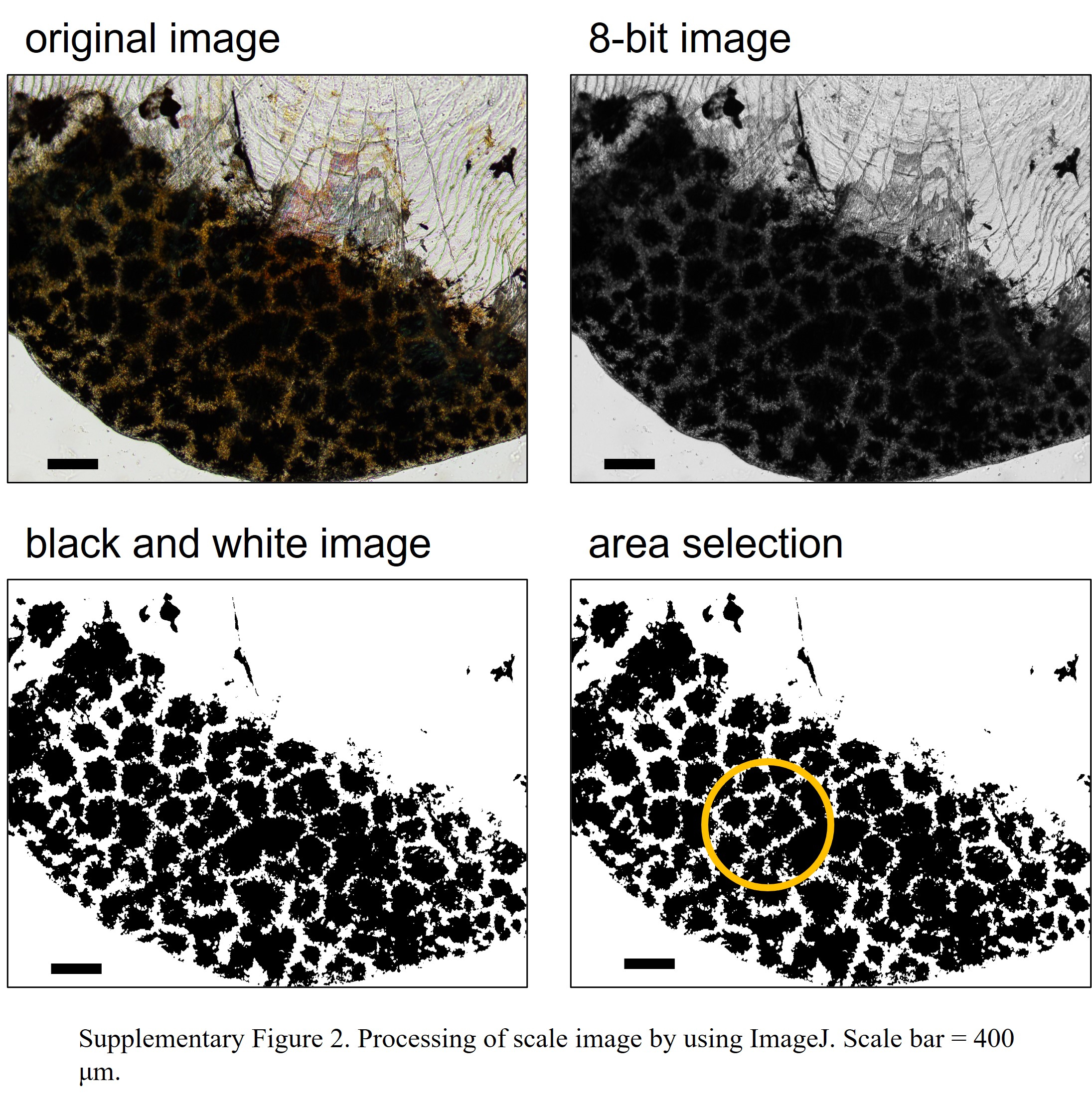

Supplement: Supplementary file 4 [file Image_2.jpeg]

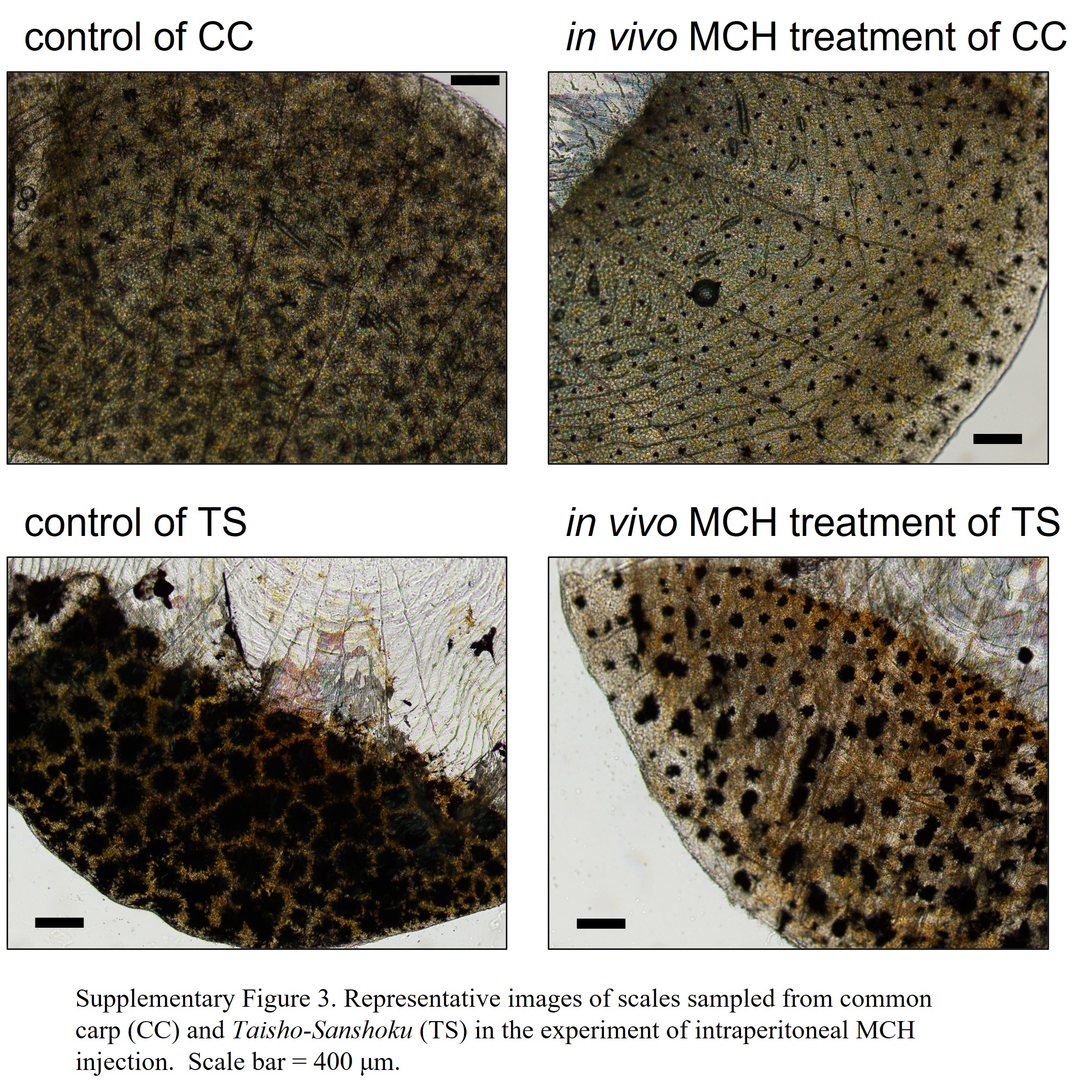

Supplement: Supplementary file 5 [file Image_3.jpeg]

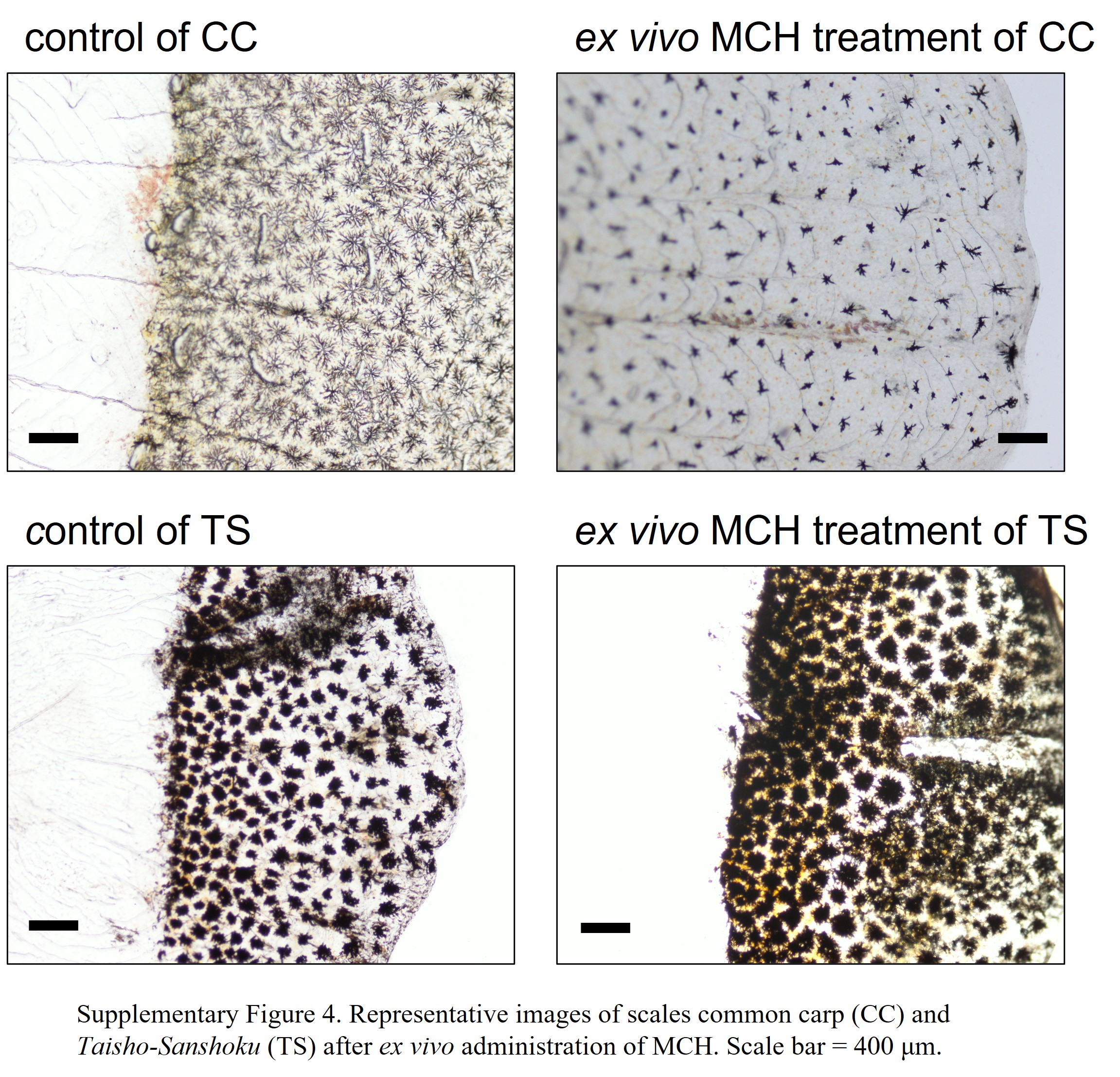

Supplement: Supplementary file 6 [file Image_4.jpeg]

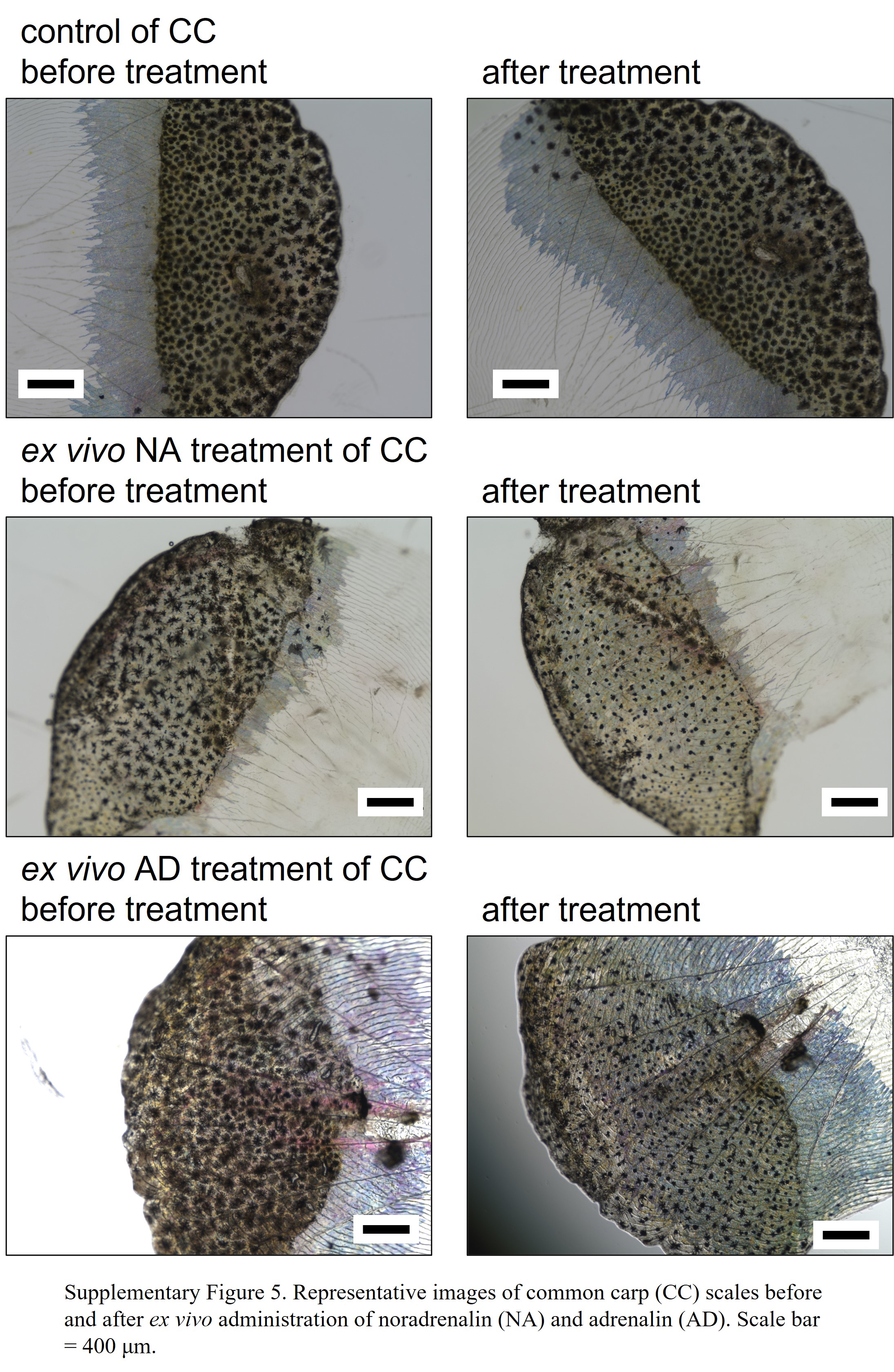

Supplement: Supplementary file 7 [file Image_5.jpeg]

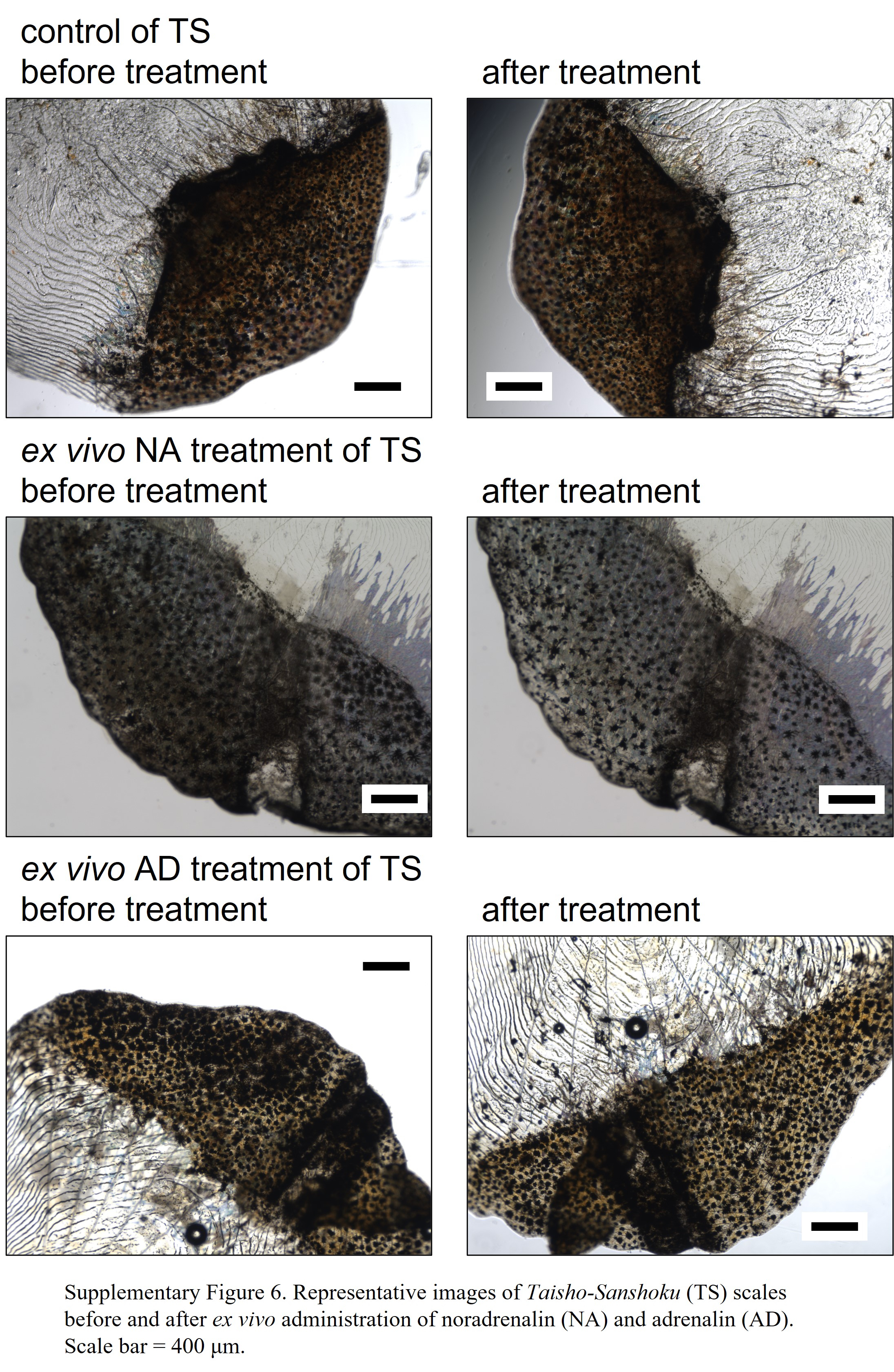

Supplement: Supplementary file 8 [file Image_6.jpeg]

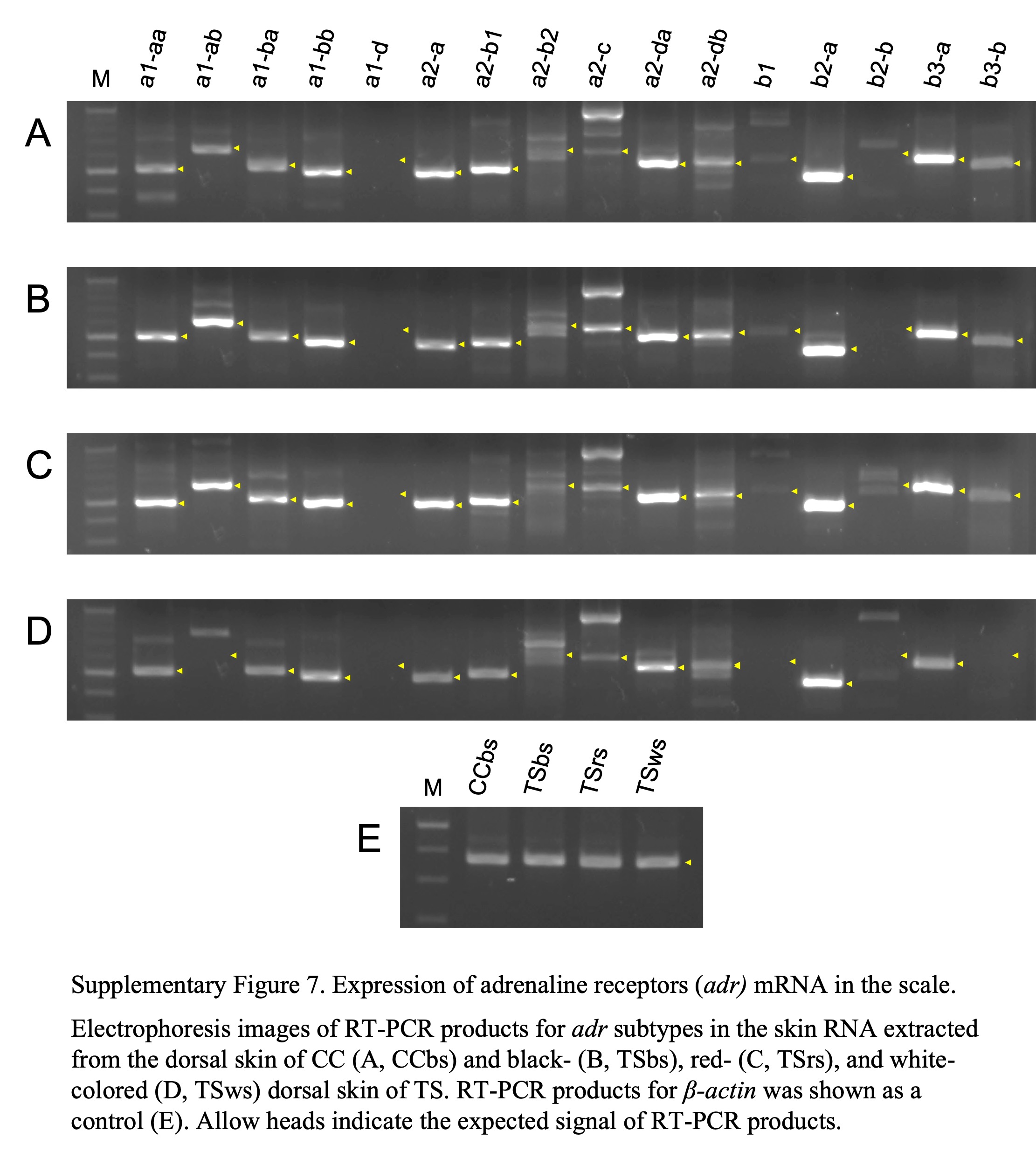

Supplement: Supplementary file 9 [file Image_7.jpeg]
